# An Intrinsically Explainable Method to Decode P300 Waveforms from EEG Signal Plots Based on Convolutional Neural Networks

**DOI:** 10.3390/brainsci14080836

**Published:** 2024-08-20

**Authors:** Brian Ezequiel Ail, Rodrigo Ramele, Juliana Gambini, Juan Miguel Santos

**Affiliations:** 1 Instituto Tecnológico de Buenos Aires (ITBA), Buenos Aires C1437, Argentina; bail@itba.edu.ar; 2 Centro de Investigación en Informática Aplicada (CIDIA), Universidad Nacional de Hurlingham (UNAHUR), Hurlingham B1688, Argentina; juliana.gambini@unahur.edu.ar (J.G.); juan.santos@unahur.edu.ar (J.M.S.); 3 CPSI—Universidad Tecnológica Nacional, FRBA, Buenos Aires C1041, Argentina

**Keywords:** XAI, BCI, EEG, P300, ALS, waveform, deep learning, CNN

## Abstract

This work proposes an intrinsically explainable, straightforward method to decode P300 waveforms from electroencephalography (EEG) signals, overcoming the black box nature of deep learning techniques. The proposed method allows convolutional neural networks to decode information from images, an area where they have achieved astonishing performance. By plotting the EEG signal as an image, it can be both visually interpreted by physicians and technicians and detected by the network, offering a straightforward way of explaining the decision. The identification of this pattern is used to implement a P300-based speller device, which can serve as an alternative communication channel for persons affected by amyotrophic lateral sclerosis (ALS). This method is validated by identifying this signal by performing a brain–computer interface simulation on a public dataset from ALS patients. Letter identification rates from the speller on the dataset show that this method can identify the P300 signature on the set of 8 patients. The proposed approach achieves similar performance to other state-of-the-art proposals while providing clinically relevant explainability (XAI).

## 1. Introduction

The area of brain–computer interfaces (BCIs) involves the quest to create an alternative communication channel between the central nervous system and a machine not based on natural pathways [1]. Recent advances have shown impressive achievements, like controlling a prosthetic limb with the mind [2,3] or a monkey playing the computer game Pong [4]. Although these outstanding developments are based on invasive electrodes, a steady improvement albeit with modest results is also being attained with noninvasive approaches, particularly through the use of electroencephalographic (EEG) signals [5].

How to harness the information from these signals? The P300 is an event-related potential (ERP), a positive deflection that appears on EEG signals around 300 ms after the onset of an unexpected stimulus. It can be cleverly used to implement a speller device that exploits the appearance of this waveform indicating the event which a person is paying attention to. BCI systems based on P300 need to identify this voltage variation from a noisy EEG signal, and by doing that they can decode the message a person is trying to convey [6,7].

In terms of potential medical applications, patients with severe cases of amyotrophic lateral sclerosis (ALS) [8] can present a locked-in state, in which they are unable to move any of their muscles to communicate. Thus, traditional alternative augmentation communication (AAC) devices [9], that harness information from any remaining muscle activity, are not effective. Creating a BCI device may be the only remaining choice to allow the person to connect with the outside world and help them to improve their life quality [10,11].

Particularly in high-stakes systems, like those for healthcare applications, any inference model based on automatic decision-making requires providing an explanation or interpretation about how the system decoded the information [12]. For ALS patients, the issue of understanding the stability of the P300 signal is paramount and goes beyond the detection of this pattern, having also clinical endpoints like aiding the understanding of neurogenerative disease [13,14].

A plethora of methods have been applied to decode EEG signals [7,15] and particularly to decode P300 components [16]. However, only a few have focused on the idea of determining the presence of the P300 ERP response based on the waveform that can be obtained from a signal plot [17,18].

Concurrently, the success of deep learning in several fields [19] has opened the possibility of using this same technique to identify the complex patterns that can be found in EEG signals [20,21,22,23,24,25]. Particularly, convolutional neural networks are successful techniques that have pushed this field ahead [26] and are often used in computer vision or speech recognition applications. Moreover, they have also been applied extensively to decode EEG signals [27,28,29,30,31,32]. Two approaches are important to note. The first is called BCINet, which is a convolutional neural network used to extract workload markers from EEG signals [33]. The other approach, called EEGNet [34], is also a convolutional neural network designed generically to process EEG data, using the convolution operation as a way to implement multiscale filter-banks [35].

Despite these advancements, the utility of DL in processing EEG signals, and healthcare information in general, is still debatable [36,37,38,39]. This is mostly due to two reasons: (1) The training steps of DL models require as many data samples as possible to achieve convergence and to generalize better, though in healthcare applications, data from patients are not readily available, and are much harder to obtain. (2) The features learned by using deep learning models face the problem that they cannot be easily understood [40], a characteristic which is often called a black box problem. Computational inference systems can have the property of being interpretableor explainable (XAI). Explainability refers to the capacity of a system to reveal the sequence of decision-making steps that the model followed throughout the inference process, while interpretability refers to the feasibility of rendering the prediction understandable to humans [41,42]. Computational inference systems that are not readily interpreted or explained are often called black box systems [43].

The work presented here circumvents this problem because the DL-CNN is used for what it is known to already perform well: decoding information from images. This decoding is based precisely on something intrinsically interpretable that we humans also perform well, detecting patterns by the visual identification of waveforms.

This proposal expands on the work performed for the dissertation [44] and follows the line of methods proposed in [18,45] and in the dissertation [46], where the waveform is also analyzed but using traditional tools from computer vision. Hence, the aim of this work is to analyze P300 signals by an intrinsically interpretable procedure which automatically detects this component from the signal plot, something that a clinician or physician can easily visually corroborate [47]. To do so, this work proposes to use deep learning (DL) techniques, particularly convolutional neural networks (CNN), which have been proven very efficient in extracting information from images, in this case images of signal plots. At the same time, this proposal tackles one of the most important issues of applying DL techniques to health information, which is the black box nature of neural network architectures [43]. It emphasizes the usage of a new form of intermediate feature engineering in terms of producing actionable analytics [48], insight data that can be extracted from the problem, and that have a certain meaning in terms of clinical knowledge. This is implemented by a pragmatic approach tailored for this particular problem where data are scarce [49].

This work proceeds as follows: Section 2 explains the details of the public dataset of ALS patients, and describes the architecture designed to process it. In Section 3, the results are expounded and compared against other similar procedures. Section 4 presents a discussion. Finally, in Section 5, conclusions, limitations, contributions and future work are highlighted.

## 2. Materials and Methods

### 2.1. P300 Experiment

**Figure 1:**
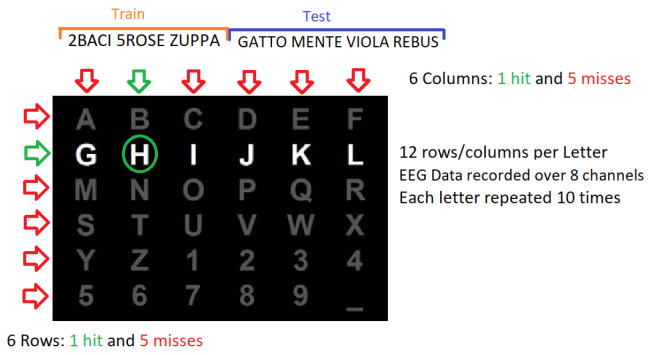
P300-speller matrix used in the ex periment. The 7 five-letter words, divided into train and testing, are shown on top. These are used in the P300 experiment for the copy-spelling task.

The dataset used in this work is the 008-2014 published under the BNCI-Horizon 2020 website [50]. The original experiment was performed by Riccio et al., 2013, where a group of eight individuals with confirmed ALS disease were tasked to spell 7 five-letter words (35 total letters) with a standard P300 matrix, which can be seen in Figure 1 (experiment details and ethical approval information available in [51]). This P300-based speller device works by flashing alternately the rows and columns of the matrix. The flashing, or letter intensification, serves as a visual stimulus that triggers the P300 response on an EEG signal segment, and by detecting it, the selected letter can be inferred from the intersection of the row and column.

Each letter spell attempt is composed of 10 flashes of the 6 rows and 10 flashes of the 6 columns of the P300 matrix. Each flash lasts for a total of 0.125 s, followed by an inter-flashing pause of the same duration. After every 120 flashes, an inter-trial pause is performed before moving to the next attempt [6].

This experiment was performed with an 8-channel EEG device (g.Mobilab, g.Tec, Austria), with a sampling frequency of 256 Hz with electrodes placed at the Fz, Cz, Pz, Oz, P3, P4, PO7, and PO8 channels according to the 10/20 international system. The reference for all the channels was the right earlobe and they were grounded to the left mastoid. The software used for implementing the speller and the processing was the BCI2000 [52]. The eight subjects were instructed to complete a copy-spelling task, which means that they had to spell a predetermined set of words that was instructed beforehand. In the original experiment, and in the approach used in this work, the objective is to use the first three words for calibration/training, while the remaining four are used for letter identification.

### 2.2. BCI Simulation

The task of decoding information from brain signals inherits practices from machine learning (ML). Nested cross-validation [53] is used in ML to reduce overfitting bias and to increase the independence from the dataset that is used as calibration. However, the brain data used in BCI are extracted from a person who is performing a task and whose signals are changing while trying to adapt to this operation. Hence, taking for granted exchangeability [54], mixing the dataset, shuffling the sessions and trials is at least a challenging assumption. BCI simulation, on the other hand, is not very well defined in BCI research, but its practice, without naming it, has been the regular approach for BCI competitions. It consists of reproducing the operational sequence that was utilized to generate the dataset. Therefore, the experiment is replicated offline using the training information to train or calibrate a classifier, and then to classify the testing signals as if they were generated at that same moment. In order to simulate a real use-case of a subject actually using the interface, the training is performed with the first letters and then used to decode the remaining letters simulating the online procedure.

### 2.3. Signal Preprocessing and Plot Generation

The original dataset was sampled at 256 Hz. We kept the same sampling frequency (i.e., without any spectral filtering). The next step involves processing the signal to enhance the signal-to-noise ratio of the P300 component. Finally, the processed signal is plotted to generate an image, which will contain the visually relevant P300 waveform.

Electroencephalographic signals are complex, non-stationary, noisy, and often contaminated by artifacts, originating either as biological sources from the human subject, from the environment, or from the recording device itself [55]. In order to detect the P300 component, it is important to maintain a high signal-to-noise ratio (SNR), which is defined as the information content between the P300 element versus all the rest. When the SNR is low, the target signal is harder to detect, since it is obfuscated by noise.

A common approach to improve the SNR is by using *signal averaging*. This technique can be applied for time-locked signals where the timing of the signal is known, the noise and signal are not correlated, the noise has a Gaussian distribution with zero-mean, and the signal is consistent if the experiment is performed multiple times [56].

A raw single-channel signal can be characterized as a time point sequence. This sequence is obtained by a digitalization process, at a certain sampling frequency $F_{s}$ which depends on the electrophysiological device [57]. The original EEG stream can be divided into segments or epochs $x_{i}\left(t\right),1\leq t\leq L$; *L* is a fixed length and rescaled to have zero-mean, which we assume meets the conditions mentioned earlier. These segments can be described as:

(1) $$x_{i}\left(t\right)=n_{i}\left(t\right)+s_{i}\left(t\right),\;1\leq t\leq L,\;i=1,\ldots ,N$$

where $x_{i}$ is the signal segment on the *i*th repetition of the trial, the flashing of a row or column, and *L* is the length of the segment. This signal segment is composed by a noise term $n_{i}$ and a timed locked signal term $s_{i}$. If we repeat the same experiment *N* times and average them, the resulting averaged segment is:

(2) $$X\left(t\right)=\frac{1}{N}\sum_{i}^{N}\left(n_{i}\left(t\right)+s_{i}\left(t\right)\right).$$

Since the noise can be considered random with a zero-mean, and the time locked signal has a similar pattern throughout the segments, we can approximate:

(3) X(t)≈S(t),

where S(t),1≤t≤L is the average of the time-locked signal segments. In this case, we have removed the noise and have a clearer signal. It should be noted that this equation can only be considered valid once *N* is large enough since we need an infinite number of repetitions to truly eliminate the noise.

One remarkable point is that when averaging independent signals and $Var\left(x_{i}\right)=\sigma^{2},\;\forall i=1,\ldots ,N$, with N>1, the variance of the averaged signal is lower than the variance of each individual $x_{i},\;i=1,\ldots ,N$:

(4) $$Var\left(\frac{\sum_{i=1}^{N}x_{i}}{N}\right)=\frac{1}{N^{2}}\sum_{i=1}^{N}Var\left(x_{i}\right)=\frac{N\sigma^{2}}{N^{2}}=\frac{\sigma^{2}}{N}<\sigma^{2}.$$

This aspect is relevant in this case because the shape of the waveform depends on the variance of the signal and the P300 experiment naturally produces a skewed distribution of segments between the classes. Hence, any classifier based on the variance, trained on this problem, could discriminate between images produced with a different number of samples, instead of detecting the difference based on the shape of the P300 component. To avoid confounding these two things, the averaged signal X(t), 1≤t≤L is normalized by applying the z-score transform [58], effectively normalizing the variance on all the signal segments from both classes.

Plotting an averaged signal segment entails a digitalization process and it produces a binary image per X(t), 1≤t≤L, with the trace representing the time-varying signal [59]. This binary image I can be constructed based on Equation (5),

(5) $$I\left(z_{1},z_{2}\right)=\left\{\begin{array}{cc} 255 & \mathrm{if}\;\left(z_{1},z_{2}\right)=\left(\gamma_{w}\;t\;,\left\lfloor \gamma_{h}\;X\left(t\right)\right\rceil +Z\right)\\ 0 & otherwise \end{array}\right.$$

where 1≤t≤L, $Z=\frac{H_{y}}{2}$, with $H_{y}=2\;\gamma_{h}\;|\max X\left(t\right)-\min X\left(t\right)|$ and $W_{x}=\gamma_{w}\;L$ being the image height and width, respectively. The symbol $\left\lfloor \cdot \right\rceil$ is the integer part operator. The coordinates $z_{1}$ and $z_{2}$ are on the horizontal and vertical axles of the image, respectively; $z_{2}$ increases from top to bottom, so the (0,0) position is on the upper-left corner of the image. The amplitude scale factor $\gamma_{h}$ and time scale factor $\gamma_{w}$ are used to determine the image size and, at the same time, the image resolution. In order to complete the trace of the signal plot, the isolated points produced by Equation (5) are connected using the Bresenham [60] algorithm, which performs a linear discrete interpolation between the pixels. This scheme produces a black-and-white plot of the signal with 255 being white and 0 black. There is one image per channel per segment.

In this work, the averaged signal segments {X(t),1≤t≤L} have a length of 800 ms sampled with a frequency $F_{s}=256$ Hz, resulting in a total of L=204 datapoints.

This generates an image where the plot fills the whole width, and leaves 25% of air on the top and bottom. The parameters $\gamma_{w}$ and $\gamma_{h}$ are scaling values that were set at 2 and 30, respectively, and *Z* is the vertical center of the image and corresponds to the position on the image where the signal is zero. As the max and min of each signal are different, the height of the generated images varies from image to image.

Finally, an image rescale is implemented using bilinear interpolation to 150×150 pixels, generating squared images. This is due to the fact that we configured the proposed neural networks to use inputs with that same dimension, and as mentioned earlier, the height of each image depends on the maximum and the minimum value of the signal. This rescaling provides a standard size for all of the signal plots.

**Figure 2:**
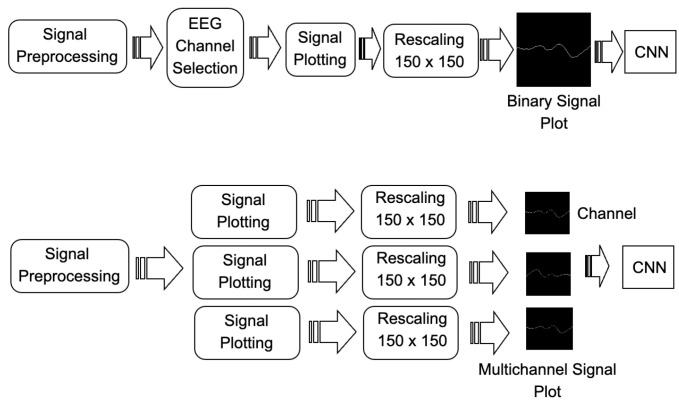
Two alternatives pipelines are used: (**top**) with channel selection, (**bottom**) bundling the information from all the channels together.

These images are used following two different pipelines, as can be seen in Figure 2. The first alternative (top of Figure 2) includes a channel selection procedure based on picking the best performing channel to plot the input image, while the second alternative (bottom of Figure 2) uses a different approach where the information from all the channels is used together at the same time to create a multichannel waveform image. This image is used as input to a CNN that will be trained to classify images containing the waveform versus those that do not.

### 2.4. Neural Network Architectures

This work proposes three neural network architectures to identify the pattern of the P300 component on the signal plot. They are based on VGG16, one of the first deep convolutional neural network architectures, which was used to win the ImageNet Large Scale Visual Recognition Challenge (ILSVR) competition in 2014 [61]. It is characterized by several layers, but at the same time, it keeps a simple structure based on the convolution operation. Convolutional neural networks are bioinspired deep learning neural networks, organized in a sequence of layers, that process an input to provide a desired output. The transition of the information from one layer to the next is mediated by the convolution operator, where the values of the convolutional kernels are the weights that are being optimized to achieve the desired output. This acts like a filter that needs to be learned. The sequence of convolutional layers is intermixed with selective decimation operations called pooling [62]. This combination of operations is particularly well suited to process visual images, in our case, visual plots of signal waveforms.

The image plot generated by the previously described procedure is used as input to each CNN for the first two proposed architectures. For the third one, the eight plots, one per channel for one segment, are bundled together in a single input.

Regarding the training, it is performed on each architecture with the first 15 letters of the speller (i.e., the first 3, 5-letter words), and the network is used to predict the remaining 20 letters (4, 5-letter words). The performance of the network is calculated by finding the row and column with the highest chance of containing the P300 signal according to the prediction of the neural network. This can be used to determine the predicted letter, which is then compared to the original expected letter from the experiment. These predictions are performed by training using the images derived from each channel separately, and then finding the channel that performs the best for the first two architectures, or combining all the images together for the third one.

#### 2.4.1. VGG16

**Figure 3:**
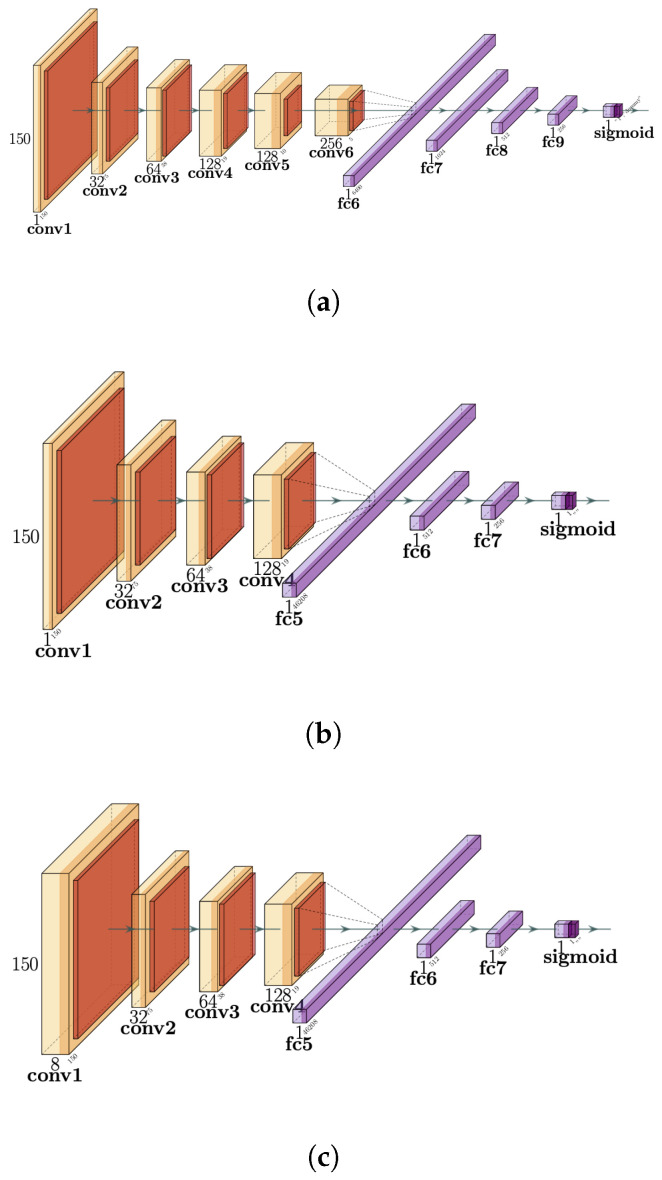
The three architectures proposed in this work. (**a**) VGG16: First version of the CNN. The input is a binary image plot of a signal of size 150×150. Then follows a set of 6 convolutional layers, followed by 4 fully connected layers, and a final layer activated using a sigmoid function. (**b**) The second, SV16 is similar to VGG16, with the same input but has 4 convolutional layers and 3 dense layers. (**c**) Finally, MSV16 has the same architecture as the SV16, but now the input layer is modified for an 8-channel input, with one binary image plotted with a signal waveform per channel. MaxPool layers are shown in dark orange.

The first version of the neural network (Figure 3a) is based on vanilla VGG16. It uses a 3×3 filter with a stride of 1 and uses padding to keep the same spatial dimensions, followed by a MaxPool layer of 2×2 size and stride of 2, and follows this arrangement of convolution and MaxPool layers all the way throughout the whole architecture, for the convolutional and MaxPool sets. The original VGG16 has 2 fully connected layers, while for this particular case, we are using 4. In the end, the last layer with only one neuron is activated with a sigmoid function to perform the binary classification. The network implementation has 6 convolutional layers with depths of 1, 32, 64, 128, 128 and 256. The stride in the MaxPool layer reduces the spatial size in each iteration, leaving a spatial size of 150, 75, 38, 19, 10, 5. After the convolutional layers, there is a dropout layer with a drop rate of 0.5, followed by a flattened layer to prepare the data for the dense layers. The 4 dense layers have a size of 6400, 1024, 512, 256, and 1 unit, with the last one having a sigmoid activation. The optimizer used for the training procedure is Adam [63], and the loss function is the mean square error (MSE). The learning rate used is adjusted to $5\times 10^{-4}$, which deviates from the recommended $3\times 10^{-3}$ [61]. Finally, the batch size is adjusted to 20. The number of trainable parameters for this model is 7, 209 or 824.

#### 2.4.2. SV16

The second approach is the Small VGG16 (SV16), shown in Figure 3b. The first change is to reduce the network size, so 2 convolutional layers and 2 fully connected layers were removed. Leaving the convolutional layer depths at 1, 32, 64, and 128, with spatial sides of 150, 75, 38, and 19. The flatten and dropout layers are kept, and then, finally, 3 fully connected layers of sizes 46, 208, 512, and 256 are added. Another addition in this version is an early stopping at 7 epochs. The remaining components of the architecture are kept unmodified, i.e., the padding, optimizer, batch size, filter size, and stride. The increase in fully connected layers brings an increase in terms of the number of trainable parameters to up to 23, 790, and 048.

#### 2.4.3. MSV16

Digital images are encoded by pixels in a square grid with a certain height and width. Grey images use one value per pixel, and this value determines the grey intensity on the screen. Color images on the other hand, use more than one value per pixel. For instance, these values can be representations of red, green and blue intensities. Furthermore, this idea could be extended to even more color channels, each representing an independent intensity, just like the colors. Particularly, convolutional neural networks are designed to analyze color images, where each color represents an independent channel of information. With the Multichannel Small VGG16 (MSV16), represented in Figure 3c, instead of selecting just one EEG channel, and training the network with the plots produced from this EEG channel, they are bundled into a single multichannel waveform image, which is the combination of the 8 plots, each one per channel acting as if it were a different color. Then the network is trained with this color image plot as input. This requires the modification of the input layer to have a depth of 8 (instead of 1), but the subsequent layers are left intact, preserving the same number of trainable parameters, 23, 790, 048.

Nonetheless, changes are required to the network architecture to cope with this change in the input data: the batch size is reduced to 6, as having a greater batch size imposes a higher memory requirement and the learning rate is readjusted to the recommended value for VGG16 of $3\times 10^{-3}$.

### 2.5. Dataset Balancing

The cognitive oddball paradigm used to trigger the P300 signal inherently has the problem that the produced dataset is unbalanced [64]. In this particular case, for each letter, there are 20 segments that belong to the hit class and 100 that do not. This skews and biases the training of the CNN. Hence, we implemented a quick and effective solution of resampling the minority class on training, which is to pick randomly the same number of samples for each class to fill the network batch size and discard the rest.

### 2.6. Software and Hardware

The code for all the experiments runs on an HP Pavillion laptop with an Intel I7 @2.8GHz processor. The available RAM is 16Gb, and the entire stack runs on CPU. No GPU was required.

The software for the signal segmentation and processing is written in Python, using a modified version of the repository EEGWave, which is publicly available on the CodeOcean platform [18]. It uses the MNE library [65] for segmenting the data and some operations with numpy [66] for signal processing procedures. On the other hand, the generation of the plot of the signals is written in C++ using OpenCV, and all the neural network architectures are created using the very efficient Tensorflow’s API for C++, based on Benny Friedman’s implementation and article [67]. The full code is public and can be found at https://github.com/shipupi/BciSift/ (accessed on 19 August 2024). For the sake of replicability [68], all the software and data for this experiment are fully available online.

## 3. Results

In this section, the results of applying different alternatives to decode the speller letters are shown. By using plain letter identification rates, meaning the percentage of correct spelled letters over the 20 available number of letters to spell, we obtain a solid metric that unequivocally reflects the capacity of each method in identifying the spelled letter directly from the brainwaves.

To compare the results of applying the proposed methods with another alternative that does not use artificial neural networks, we utilize the algorithm presented in [45], based on the scale invariant feature transform (SIFT). This method can find distinctive image features using a histogram of oriented gradients from pixel intensities. In addition, it has the advantage of being invariant under scale and affine transformations [69].

**Figure 4:**
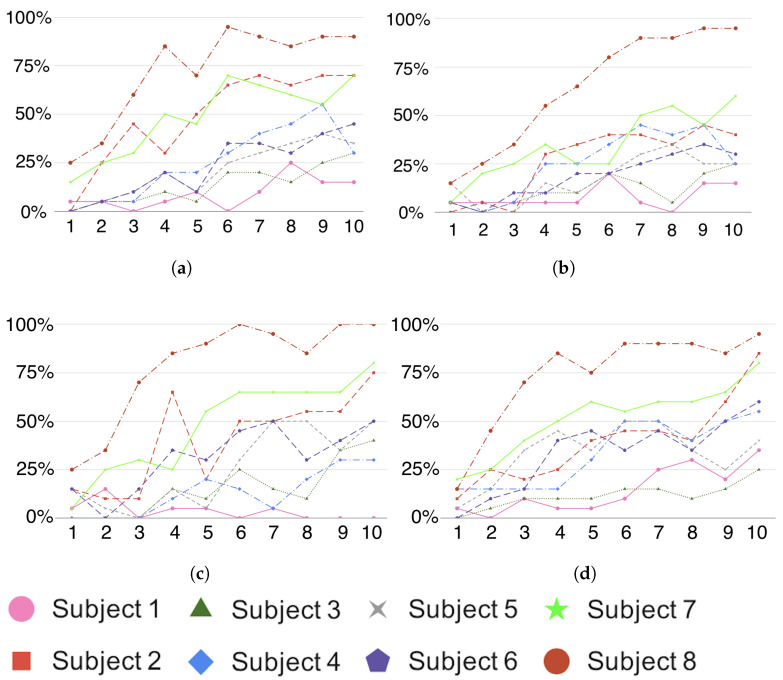
Side-by-side comparison of letter identification rate (*y*) per number of letter intensifications (*x*) for all the three architectures proposed in this work and one more for comparison: (**a**) VGG16, (**b**) SV16 and (**c**) MSV16, (**d**) SIFT method [45].

Figure 4 shows the results of computing the letter identification rate, by measuring success in the letter prediction. The x-axis represents the increasing number of letter intensifications that are used to calculate the averaged ERP response (i.e., using more information). Figure 4a–c represent the results obtained by using VGG16, SV16 and MV16, respectively. For the sake of comparison, results obtained from the SIFT method [45] are shown in Figure 4d.

**Figure 5:**
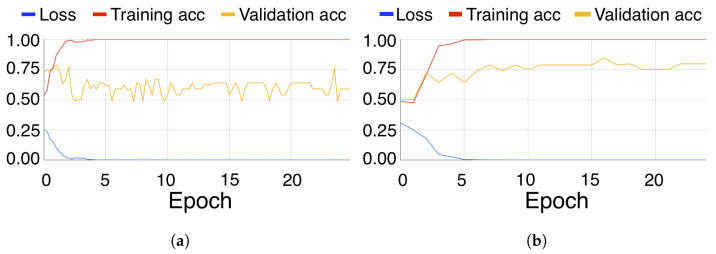
Learning curve, showing loss, training accuracy and validation accuracy of subject 5 on channel PO8 with an intensification level of 4 using (**a**) VGG16 and (**b**) SV16.

It can be observed that the three networks were successfully trained with the generated plots. The learning curve for subject 5 of the dataset at an intensification level of 4 can be seen in Figure 5. Figure 5a,b show the loss value, training and validation accuracy of using VGG16 and SV16, respectively. Comparing both, the accuracy value for the validation set is much higher for the SV16, while the accuracy value for the training set remains at or close to 100%.

Letter identification success rates for 10 letter intensifications are shown in Table 1 for each subject from the experiment dataset. Stepwise linear discriminant analysis (SWLDA) values are based on the algorithm reported and used by Riccio et al., 2013 [51]; SIFT are the values obtained by using SIFT descriptors. The table includes results using the vanilla SVM method [45], BCINET [33] and EEGNET [34].

## 4. Discussion

Overall, the obtained results show that all three proposed architectures achieve a success rate while identifying spelled letters, which is comparable to other similar published works on this same dataset. Moreover, success rate values are consistently increasing along the intensification level. We did not find any significant difference between all the eight methods listed in Table 1 (Quade Test, F=1.76, p=0.11) [70].

Nonetheless, there are important points to highlight. First, the advantage of using an already established model like VGG16 is that it reduces the number of hyperparameters that need to be tinkered with, as all the filters and strides are known to be effective for processing images. Finding a suitable architecture and its parameters is a daunting task. It is clear from the literature that there is no certainty as to which hyperparameters to use when dealing with information from EEG signals [71].

Another aspect to note is that the three proposed architectures required some data balancing mechanism in place, otherwise the neural network was unsuccessful in converging (i.e., not reducing successfully the training error). This is also found in similar works, either by subsampling the majority class or by resampling the minority class on training [71].

Regarding the VGG16 architecture, the main issue we have found is overfitting. The training sets are being learned perfectly, reaching a 100% accuracy, but the accuracy on the validation set does not increase and oscillates around 60%. The SV16 version attempted to address this issue by adding early stopping and reducing the number of layers, as shown in Figure 3b.

**Figure 6:**
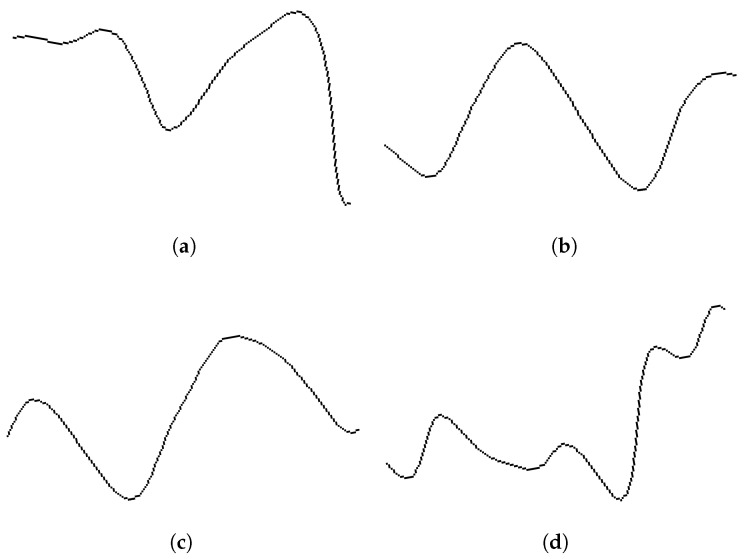
Images of P300 waveforms (**a**,**c**) from the first 0.700 ms of stimulus-locked segments of channel Cz of subject 8, obtained by averaging the signal segments triggered from 5 intensifications. Images (**b**,**d**) are the obtained shape when this waveform is not present.

The biggest increase in performance in this work is the MSV16 version, with the inclusion of all the plots of the EEG channels into a single multichannel waveform image plot. This third and final version of the network included both the improvements made in SV16, as well as the addition of multi-channel classification, and a smaller batch size. Additionally, this approach can help to understand spatial asymmetries in the signal, which can have clinical implications. This last version showed an accuracy increase in six out of eight subjects over the other two versions. It surpasses SIFT in three out of eight subjects and performs equally in one. It does, however, seem to underperform on subjects 1 and 4 compared to earlier versions and SIFT. But it also reaches a 100% accuracy on subject 8, as was the case for EEGNET. Also, by looking at the accuracy per intensification level, subject 8 achieved over 90% accuracy in only five intensifications, meaning that a robust speller could be established with a much faster transmission rate. This can also be visually seen in Figure 6 where a similar shape from images (a) and (c) is what the neural network is actually identifying, what it is actually seeing, the shape of the P300 waveform. The best improvement was that it allowed the combination of all the different EEG channels to work together, achieving a higher performance than each channel separately [37]. This tendency could potentially be greater for EEG signals recorded with more than eight channels.

The results of the three approaches show clearly the importance of the signal averaging procedure in increasing the signal-to-noise ratio (SNR). On the other hand, the downside of signal averaging is that repetitions of the experiment take more time, thus slowing down the information transfer rate (ITR). As BCI devices are communication systems, ITR is an important measure of performance [72], with units in bits-per-second (bps) and a method to measure the advancement of this technology. Out-of-the-box BCI systems could obtain very low transfer rates, of around only 5 bits per minute or 0.08 bps [73]. For instance, the system proposed in this work, using the MSV16 version, could achieve an ITR of 0.6 bps, at 5 intensifications per letter. More sophisticated systems, tailored for a specific group of patients using brainwaves in non-invasive SSVEP Speller can achieve 13 bps [74]. BCI systems have a wide inter-subject variability, so there are works reporting faster rates but their generalization must be carefully considered [75]; thus, it may be difficult to reach the 13 bps value for every person and even for the same person in different settings and across sessions. Invasive systems with implanted electrodes are faster, reaching from 5 up to 15 bps or even more [4]. Some specific use-cases require a very high bandwidth (>15 bps), for instance, the one needed to control all the degrees-of-freedom (DOF) of an arm prosthesis [2,76,77]. For reference, keyboard typing is 16–20 bps (200 words per minute), while speaking is 39 bps [78].

**Figure 7:**
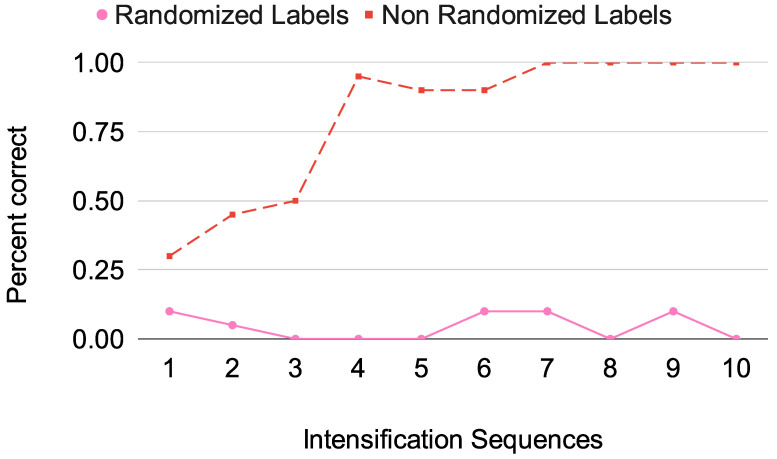
Accuracy of MSV16 on subject 8; the red dotted line shows a normal prediction using real labels on the training set, while the pink line shows the predictions of the CNN by training it with randomized labels on the training set. We can see that the accuracy on the pink line hovers around the chance level of 3%.

Finally, control experiments were performed to ensure the validity of the obtained results. First, while performing the signal averaging procedure, the standard deviation of the averaged signal segment X(t) is calculated, and it is asserted that it is indeed lower than the one for each signal segment $x_{i}\left(t\right)$. Additionally, as long as more segments are used, it is verified that it is progressively reduced. To corroborate that the obtained results are not due to chance, we ran the experiment on all the subjects but with all the labels randomized on the training dataset. The expected outcome of this procedure is that the predictions for the letters are reduced to the chance level of 1/36 ≈ 3%. This is a procedure inspired by the Boruta feature selection [79]. We can see this in Figure 7, where the accuracy of the prediction of the MSV16 network with random labels on subject 8 is oscillating around the chance level threshold, while the accuracy without randomizing the training labels goes back to the reported value. This shows that there is indeed a generalization being performed by the neural network.

Extensive work has been conducted about XAI to develop methods that aim to alleviate the black box nature of DL models [80]. CNN feature maps [81,82] are a popular approach that enables some form of explainability by analyzing decision regions in the hidden layers of the network and inferring what their contribution is to the final decision. However, they create an even wider gap that complicates the understanding and communication from the human perspective [83], and may hamper even further trust in the system [84].

Moreover, there is an existing correlation between the stability of the P300 signal pattern and indexes associated with ALS disease prognosis [13,14,46]. For instance, the images of P300 waveforms shown in Figure 6a,c correspond to a patient where it was verified that the obtained P300 signal is stable [46]. This is also what the CNN network detected through higher letter identification rates. Additionally, quantitative parameters, like the amplitude or latency of the ERP, can be directly extracted from those images themselves, providing a straightforward way to perform the automatic analysis and the qualitative visual analysis of the waveforms. Furthermore, in a previous work [46], where the waveform was analyzed by the SIFT method, we performed exactly this same experiment with a control group of healthy individuals and we found no evidence of significant changes in terms of the achieved performance on the experiment between the group of healthy volunteers and this group of ALS patients.

We believe that the approach proposed here could eventually be a feasible tool for clinical settings that aim to detect ERP abnormalities in an automated way, connecting the reasons for the performance of this deep learning detection procedure with the shape of the ERP waveform. In particular, there is evidence in the literature that ERP abnormalities in the waveforms can be detected in ALS patients in early stages of the disease without major cognitive deficits [14].

## 5. Conclusions

The purpose of this work is to provide an intrinsically explainable method for convolutional neural networks to identify the P300 signal pattern. This can aid in understanding clinical insights that are particularly relevant to ALS patient populations. It is important to remark that the proposed method provides a straightforward way for a neural network to provide explainability. By trying to process the information in a similar way that humans deal with this information, actionable analytics can be extracted and are ready to be used in the problem at hand. We believe it is highly desirable to offer a human-centered approach to explainability, emphasizing insights that could be understood by professionals and clinicians and not the other way around [85]. We force the automation to talk in human terms.

Additionally, we have shown that the obtained performance is at the same level as other methods that lack these XAI characteristics. Moreover, this approach is very general, because it can be used to decode any signal that can be represented in a plot, and the discriminating power can be encoded by the waveform inside the plot.

One of the reasons why deep learning techniques are highly successful is because they can produce tools upon which other solutions can rest. Using the original structure of VGG16 as a baseline foundation optimized the effort involved in the time-consuming and iterative process of optimizing hyper-parameters and network architectures [86].

One aspect to specifically remark on is that this procedure combines information from different channels at the same time, which perfectly matches what may be appropriate for CNN. We have verified that this approach generates better results than those obtained with signal plots images that only reflect the information from just one channel. And even though the data used are scarce for each subject, the CNN was able to obtain enough samples to be trained to effectively discriminate the P300 ERP.

Several limitations and considerations need to be taken into account. First, the original paper where this dataset was published, Riccio et al. [51], did not describe the level of cognitive impairment of each participant. In Kellmeyer et al. [14], through an extensive review of ALS biomarkers in the context of EEG studies, it was verified that there is evidence of abnormalities of ERP responses and alterations in the waveforms regardless of the level of cognitive impairment scored for each patient. Nonetheless, the influence of any level of cognitive impairment as a possible confounding factor in the ERP response cannot be neglected. Another technical limitation is that this method relies on signal averaging to obtain a meaningful pattern that can be detected by analyzing the shape of the signal. This is reflected in the low success rate in letter identification obtained for only one signal intensification. The application of this method requires that the information is encoded in the waveform of the signal. Concordantly, there is already vast established knowledge in terms of EEG waveform shapes that can be explored further with potential applications of this proposed method.

## Figures and Tables

**Table 1:** Letter identification rates in percentages for SWLDA, SVM, EEGNET, BCINET, SIFT, VGG16, SV16 and MSV16 for the public dataset [51].

Subject	SWLDA	SVM	EEGNET	BCINET	SIFT	VGG16	SV16	MSV16
1	45	40	50	45	35	15	10	0
2	30	50	30	55	85	70	50	75
3	65	55	70	65	25	30	30	40
4	40	50	60	55	55	30	40	30
5	35	45	40	45	40	35	50	50
6	35	70	60	75	60	45	40	50
7	60	35	90	80	80	70	65	80
8	90	95	100	95	95	90	95	100

## Data Availability

The data used in this work are the dataset 008-2014 publicly available from BNCI-Horizon 2020 website: https://bnci-horizon-2020.eu/database/data-sets (accessed on 19 August 2024).
